# A patient with femoral osteitis fibrosa cystica mimicking bone neoplasm: a case report

**DOI:** 10.1186/s12891-022-05274-0

**Published:** 2022-04-04

**Authors:** Xiao-Long Xu, Cui-Ping Yang, Sheng-Jun Lu, Hong Pei, Shun-Guang Chen, Quan-Ming Liao

**Affiliations:** 1 Department of Orthopedic Surgery, Jingzhou Central Hospital, The Second Clinical Medical College of Yangtze University, No. 26, Chuyuan Ave, Jingzhou District, Jingzhou, Hubei 434020 People’s Republic of China; 2 Department of Pathology, Jingzhou Central Hospital, The Second Clinical Medical College of Yangtze University, No. 26, Chuyuan Ave, Jingzhou District, Jingzhou, Hubei 434020 People’s Republic of China

**Keywords:** Case report, Hyperparathyroidism, Parathyroid adenoma, Osteitis fibrosa cystica

## Abstract

**Background:**

Osteitis fibrosa cystica is a rare, benign and osteolytic lesion attributed to hyperparathyroidism. The high level of parathyroid hormone cause rapid bone loss.

**Case presentation:**

The patient is a 50-year-old male complaining of severe and persistent pain in the right knee joint. Imaging studies were suspicious for a benign tumor in the right distal femur. Biopsy under CT guidance showed numerous osteoclast aggregation and hemosiderin deposition around the bone trabeculae. Blood tests disclosed significantly elevated parathyroid hormone, serum calcium, serum alkaline phosphatase. Parathyroid ultrasonography and CT scan showed a solid mass in front of the trachea at the thoracic entrance plane. After resection of the mass, the clinical symptoms were relieved and the radiological results were significantly improved, which further confirmed the diagnosis.

**Conclusions:**

Metabolic diseases-associated bone lesions require a comprehensive diagnosis of multiple inspection items. An interprofessional team approach to the diagnosis and treatment of osteitis fibrosa cystica will provide the best outcome.

## Background

Osteitis fibrosa cystica (OFC) is a a rare, osteolytic and nonneoplastic metabolic bone disease [1]. The activation of osteoclast in OFC is due to increased endogenous parathyroid hormone (PTH) [2]. Hyperparathyroidism is a common endocrine disorder characterized by elevated blood concentration of parathyroid hormone (PTH) and hypercalcemia, usually due to a benign adenoma. Primary or secondary hyperparathyroidism is usually asymptomatic, however, severe forms may be diagnosed by lesions of target organs such as the skeletal system and the kidneys. Among patients with hyperparathyroidism, about 20% develop kidney stone disease [3]. Osteitis fibrosa cystica can occur as solitary or multiple lesions in any bone, most often in the mandible, pelvis, ribs and long bones [4, 5]. The most typical skeletal manifestation of hyperparathyroidism were loss of cortical bone and trabecular remodeling due to osteoclast activation. OFC can be associated clinically with bone fractures, skeletal deformities and bone pain [6]. At present, osteitis fibrosa cystica, though rare, was easily misdiagnosed. Due to its histological and radiological features, it is often misdiagnosed as a bone tumor [7]. We report a case of primary parathyroid adenoma with OFC that presented clinically and radiologically as an aggressive metabolic bone disease.

## Case presentation

**Fig. 1:**
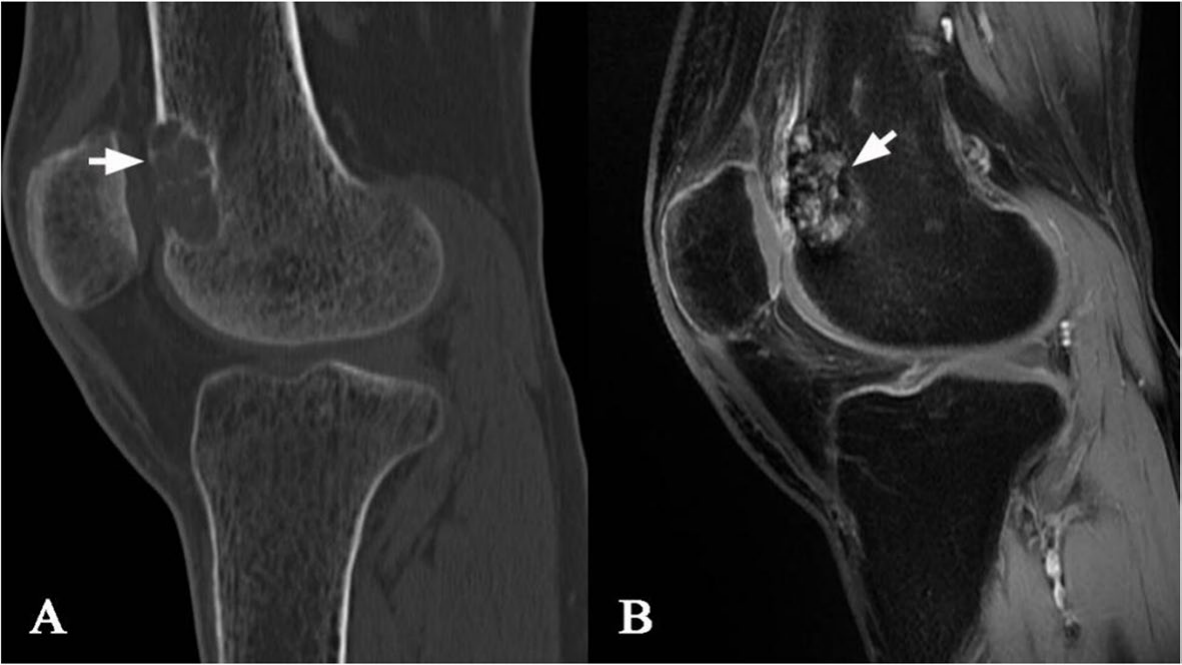
CT scan showing cyst-like bone lesion at the distal part of femur with destruction of bone cortices, soap bubble like inner structure (**a**). Contrast-enhanced magnetic resonance of the knee joint showing heterogeneous enhancement and clear boundary (**b**)

**Fig. 2:**
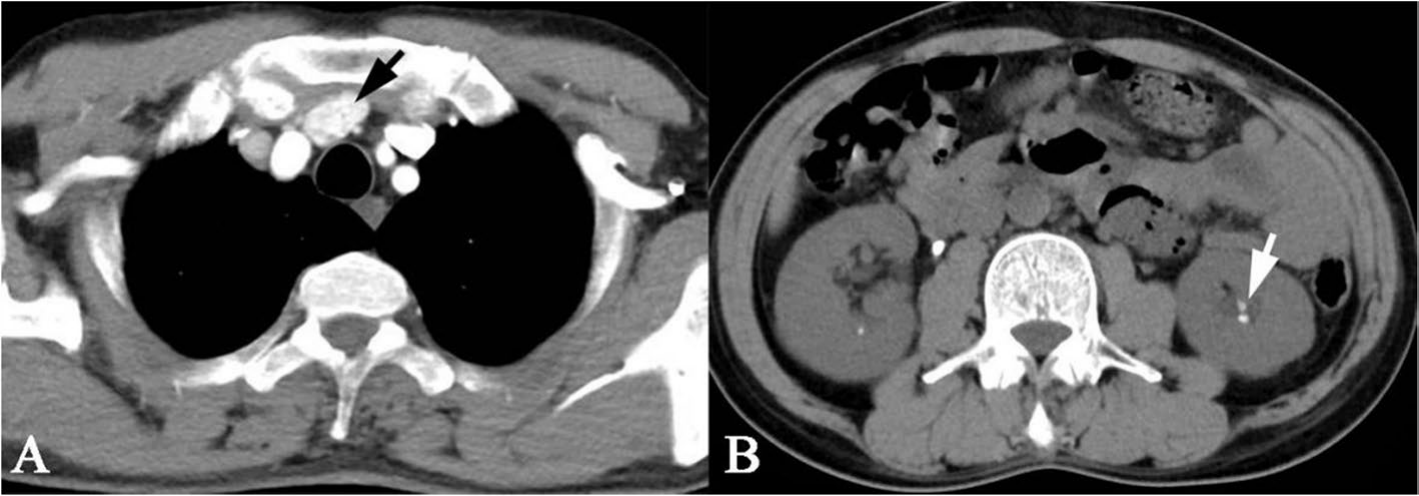
Contrast-enhanced CT scan of the parathyroid showing a solid mass in front of the trachea at the thoracic entrance plane with heterogeneous enhancement and clear boundary (**a**). CT scan of the kidney showed renal calculus formation (**b**)

**Fig. 3:**
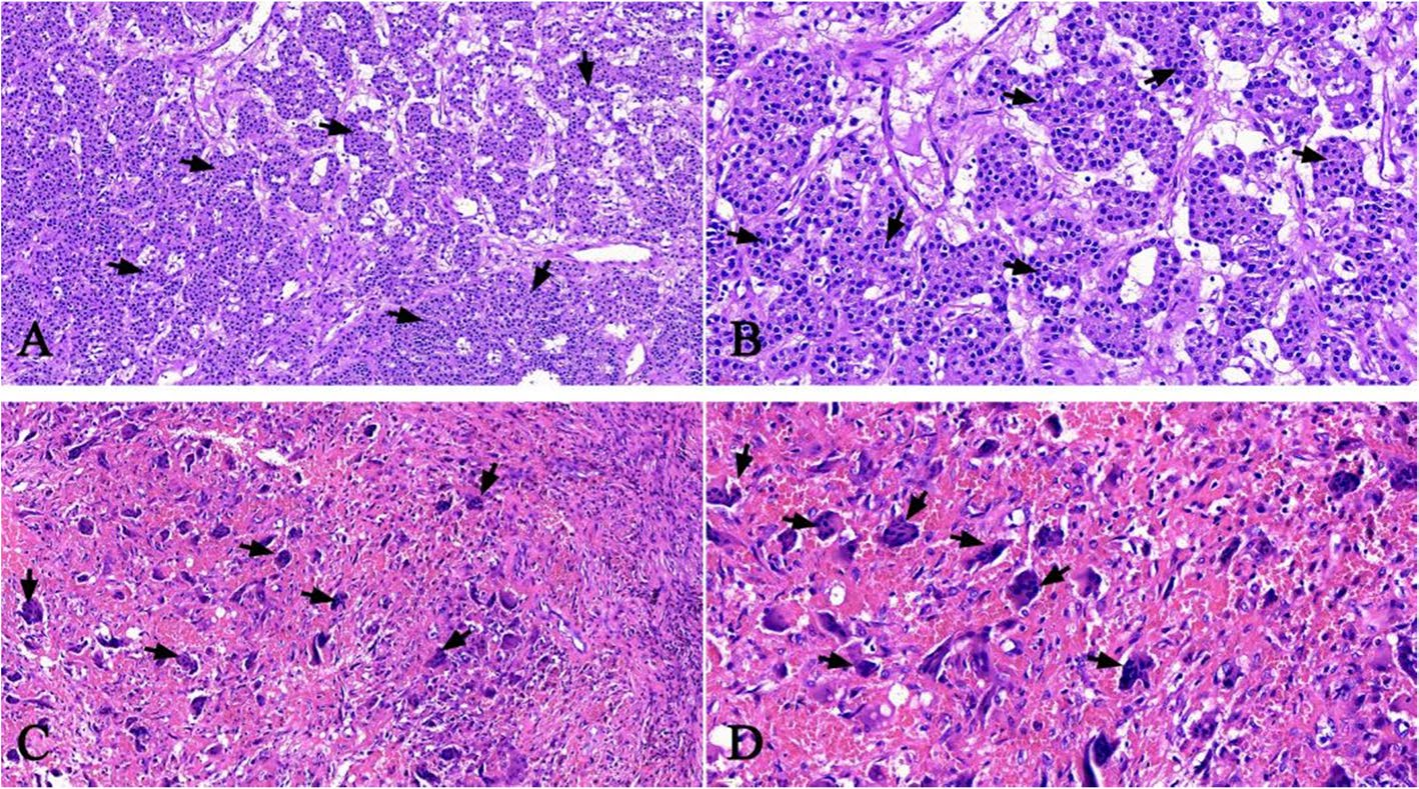
H&E stain showing parathyroid adenoma (**a**). **b** The parathyroid adenoma showing chief cells. **c** and **d** Right distal femur biopsy showing unusual high number of osteoclast like giant cells and disordered trabeculae

A 50-year-old male presented to our institution for pain in the right knee joint. The pain gets worse during exercise and relieved at rest. Physical examination was unremarkable. CT scan showed cyst-like bone lesion at the distal part of femur with destruction of bone cortices, soap bubble like inner structure, sclerotic rim and no periosteal reaction (Fig. 1). Other CT scans showed kidney stones in this patient (Fig. 2). Contrast-enhanced magnetic resonance imaging of the knee joint showed a heterogeneous enhancement and clear boundary  (Fig. 1). There was no soft tissue mass around the lesion. Biochemical blood tests revealed high calcium concentration (3.63 mmol/L, normal values 2.11–2.52 mmol/L) and low phosphorus concentration (0.5 mmol/L, normal values 0.85–1.51 mmol/L). The result tended to be a lytic bone tumor. A bone biopsy was performed under CT scan guidance, the sample was sent for histopathologic examination. Histopathologic examination showed bone lesion composed of remodeled bone trabeculae with scattered groups of multinucleate giant cells of the osteoclast type with areas of hemorrhage and hemosiderosis. There was no evidence of malignancy in the biopsy sample (Fig. 3).

In order to verify hypercalcemia caused by hyperparathyroidism, parathyroid ultrasound and parathormone (PTH) were also performed. The concentration of PTH in serum is elevated (2034.6 pg/mL, normal values 12.0–88.0 pg/mL). Parathyroid ultrasound revealed a 3.6 × 1.6 cm solid mass in front of the trachea at the thoracic entrance plane. Contrast-enhanced CT scan of the parathyroid neoplasm showed heterogeneous enhancement and clear boundary (Fig. 2). Combined with the above results, we considered that bone lesion was related to parathyroid neoplasm.

**Fig. 4:**
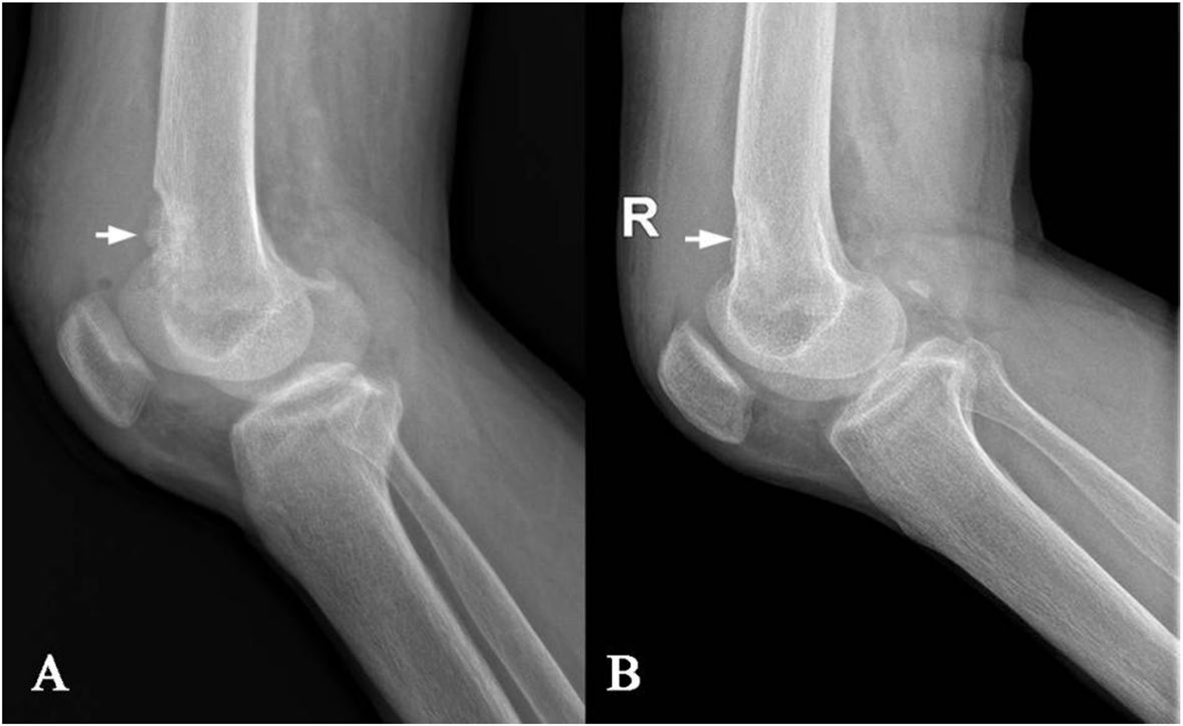
Plain radiograph of the patient showing a osteolytic lesion at the right distal femur (**a**). Radiograph 6 months (**b**) after resection of the parathyroid adenoma showing a markedly increased in mineralization and almost complete healing of the osteitis fibrosa cystica

After resection of the parathyroid adenoma, the patient no longer complained from knee joint pain and imaging performed six months later showed significant improvement, with reduction of the bone lesion dimensions and complete reconstruction of bone cortices (Fig. 4).

## Discussion and conclusion

Osteitis fibrosa cystica (OFC) is a disorder involving the bone which is resulted from excessive production of parathyroid hormone (PTH) due to hyperparathyroidism. Hyperparathyroidism might be due to parathyroid adenoma (up to 85% of cases), parathyroid hyperplasia, parathyroid carcinoma and hereditary factors [8].

Biologically, PTH induces osteoclast activity which results in abnormal structural changes and bone remodeling including osteopenia, subchondral resorption, subperiosteal bone resorption and focal lytic lesion [9]. Up to 5% of hyperparathyroidism cases develop OFC. In clinical, OFC is usually diagnosed late because they are asymptomatic unless the disease reaches advanced stages and causes clinical symptoms such as pain or deformity [10]. OFC is a metabolic and non-neoplastic lesion but sometimes may also be misdiagnosed as a malignant lesion. It can also be misdiagnosed for a metastatic disease based on the radiological results, such as multiple scattered osteolytic lesions [11].

As for the imageological diagnosis, CT showed cyst-like bone destruction, monocystic or polycystic, clear boundary and no periosteal reaction.The MRI examined no soft tissue mass formation around OFC which is a relatively characteristic phenomenon. OFC also often coexist with subperiosteal bone cortical resorption, subchondral bone resorption. Based on imaging manifestations, OFC needs to be identified from giant cell tumor of bone, aneurysm-like bone cysts, plasmacytomas. Pathological manifestations of the lesions surface is brown, formed by different size of the cysts. The cystic wall is fibrous tissue. When necrosis or hemorrhage occurred in the cystic cavity, large numbers of multinucleated macrophages and hemosiderin-rich phagocytes appeared. Osteoclasts showed a sheet-like distribution. Inhomogeneous cancellous bone trabeculae was surrounded by osteoblasts.

The diagnosis of OFC may be challenging. In clinical practice, patient’s symptoms are due to bone softening and hypercalcemia, which might include bone mass or fractures, kidney stones, peptic ulcer, weight loss, nausea and loss of appetite. However, these symptoms lack the specificity. In addition to the histological and radiological findings, biochemical tests including PTH, serum calcium, alkaline phosphatase are also very important to ensure a correct OFC diagnosis.Treatment of osteitis fibrosa cystica starts with the management of hyperparathyroidism, such as parathyroid adenoma resection. After parathyroid adenoma resection, the majority of bone disorders caused by OFC will be resolved.

In summary, in current practice, skeletal manifestations of hyperparathyroidism are less common than in the past. OFC as a kind of metabolic diseases-associated bone lesion is easily misdiagnosed as bone tumor according to radiological and pathological characteristics. Serum calcium and PTH should be conventionally checked in patients with suspected metabolic bone disease. Elevated parathyroid hormone levels and hypercalcemia are indicative of OFC and complement the radiological and histological results to further confirm the diagnosis. If treated properly, OFC is reversible.

## Data Availability

The authors declare that data supporting the findings of this study are available within the article.
